# Cellular and molecular responses of *Cryptococcus* to brilacidin

**DOI:** 10.1128/spectrum.00138-26

**Published:** 2026-06-15

**Authors:** Barbara T. Bezerra, Daniel A. Mellon, Cássia M. Souza, Rafael F. Castelli, Amanda C. Camillo-Andrade, Marlon D. M. Santos, Paulo C. Carvalho, Marcio L. Rodrigues, Haroldo C. de Oliveira

**Affiliations:** 1 Instituto Carlos Chagas, Fundação Oswaldo Cruz (Fiocruz) 169688 , Curitiba, Brazil; 2 Analytical Biochemistry and Proteomics Unit, Instituto de Investigaciones Biológicas Clemente Estable, Institut Pasteur de Montevideo 113067, Montevideo, Uruguay; 3 Instituto de Microbiologia Paulo de Góes, Universidade Federal do Rio de Janeiro 28125, Rio de Janeiro, Brazil; 4 Department of Microbiology, Immunology, and Parasitology, Discipline of Cellular Biology, Federal University of Sao Paulo (UNIFESP) 28105, Sao Paulo, Brazil; Institut Pasteur, Paris, France

**Keywords:** cryptococcosis, *Cryptococcus neoformans*, *Cryptococcus deuterogattii*, brilacidin, antifungal activity, antifungal drugs synergism, biofilm, proteomics

## Abstract

Cryptococcosis is a systemic mycosis caused by *Cryptococcus neoformans* and *Cryptococcus deuterogattii*. Available therapeutic options are limited and face several challenges, including toxicity, the emergence of resistant strains, high costs, and restricted distribution. In this study, we explored the cellular and molecular responses of *Cryptococcus* spp. to brilacidin, a recently characterized fungicidal agent with promising potential for the treatment of cryptococcosis. Brilacidin exhibited inhibitory activity with a minimum inhibitory concentration of 2.5 µM and demonstrated a high selectivity index and low cytotoxicity in murine macrophages. Synergy assays revealed that brilacidin potentiated amphotericin B activity, reducing its effective dose up to eightfold without increasing cytotoxicity. Morphological analyses by scanning, confocal, and transmission electron microscopy expanded previously described effects of brilacidin and revealed profound alterations in capsule structure, plasma membrane integrity, and intracellular organization. Brilacidin also inhibited *C. neoformans* biofilms in a concentration-dependent manner, accompanied by major structural disruption. We investigated the molecular response of *C. neoformans* and *C. deuterogattii* to brilacidin by quantitative proteomics, which revealed species-specific responses. Brilacidin primarily affected proteins involved in sterol metabolism, oxidative stress, and cell wall biosynthesis. These results deepen our understanding of the anti-*Cryptococcus* effects of brilacidin and reinforce the potential of this drug as a highly promising antifungal agent for the treatment of cryptococcosis.

**IMPORTANCE:**

Cryptococcosis is a life-threatening fungal disease that mainly affects immunocompromised individuals and remains difficult to treat due to toxic drugs, limited therapeutic options, and the emergence of resistance. This study deepens the understanding of brilacidin as a promising antifungal candidate against the causative agents of cryptococcosis. Brilacidin exhibits potent fungicidal activity at low concentrations with minimal toxicity to host cells and enhances the efficacy of amphotericin B, allowing for reduced doses that may mitigate adverse effects. Furthermore, brilacidin disrupts key fungal structures, including the capsule and biofilms, and induces distinct cellular stress responses. By providing mechanistic and translational insights, this work supports the development of safer and more effective therapeutic strategies for this neglected and often fatal fungal infection.

## INTRODUCTION

Cryptococcosis, a systemic mycosis caused by fungi of the genus *Cryptococcus*, is among the most fatal fungal infections and is responsible for nearly 200,000 deaths annually (1). Transmission occurs through the inhalation of spores or small fungal cells. Once inhaled, the infection primarily affects the respiratory system and, in severe cases, can disseminate to the central nervous system, resulting in cryptococcal meningitis (2–4). Clinical manifestations vary depending on the host’s immune status; however, it is well established that immunocompromised individuals—particularly those with HIV/AIDS—are more susceptible to severe disease progression (4–6). Antifungal agents such as amphotericin B (AmB), flucytosine, and fluconazole are typically employed to manage cryptococcosis (7, 8). However, several challenges can compromise treatment success, including high costs, limited drug accessibility, the emergence of multidrug-resistant strains, and the potential for toxicity and adverse side effects (9–12). Thus, the development of new antifungal therapies is a crucial component of successful cryptococcosis management.

In recent years, our research group has implemented a screening program utilizing compound libraries to identify novel antifungal agents. Through this methodology, several promising compounds were identified (13–16). In our most recent study using this approach, we identified brilacidin as one of these candidates, showing activity against *Cryptococcus neoformans, Cryptococcus deuterogattii,* and *Candida auris* (13), and, concurrently, Dos Reis et al. and Diehl et al. reported the antifungal potential of this compound (17, 18), showing that *in vivo* treatment with brilacidin (5 mg/kg) generated a significantly decreased lung infection in mice lethally infected with *C. neoformans*, as evidenced by a reduction of 34.5% of lung CFUs (17). However, several questions addressed in this study differed from those under investigation in our laboratory, providing complementary perspectives that together offered a more comprehensive understanding of how brilacidin affects *Cryptococcus*. Consequently, we directed our efforts toward extending the characterization of the anti-cryptococcal activity of brilacidin.

Given the urgent need for new therapeutic antifungals, this study aimed to characterize multiple responses of *C. neoformans* and *C. deuterogattii* to brilacidin. Our analyses included the effects on biofilm formation, synergism with AmB, morphological alterations, and molecular responses analyzed by proteomics. Our results expand our knowledge of how this antifungal drug affects two major human pathogens.

## RESULTS

### Antifungal efficacy versus selectivity of brilacidin

**Fig 1:**
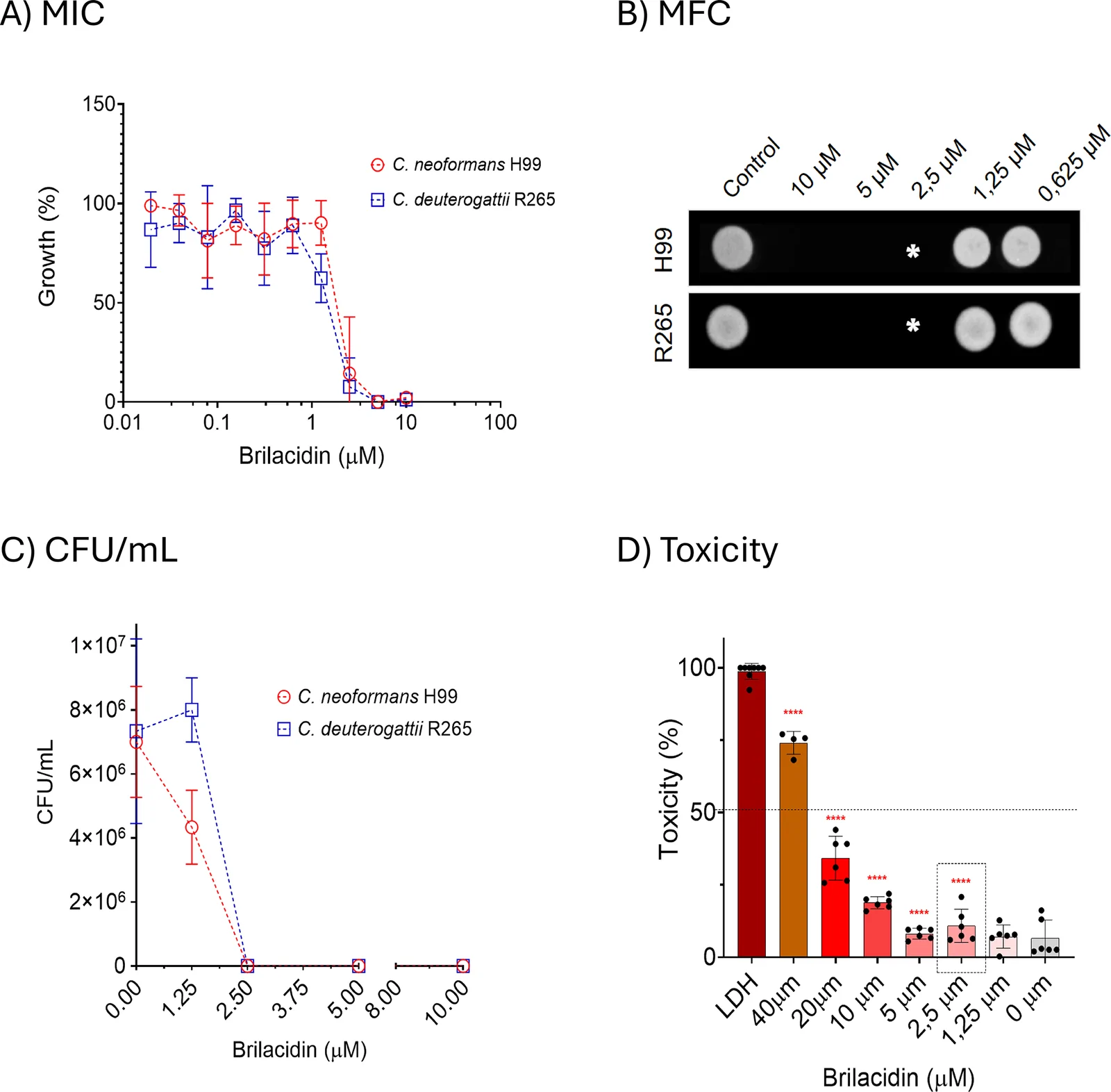
Antifungal activity and cytotoxicity profile of brilacidin against *C. neoformans* H99 and *C. deuterogattii* R265. (**A**) Determination of the MIC of brilacidin. Fungal growth inhibition was measured over a concentration range of 0.019 µM–10 µM. The MIC was defined as the concentration at which growth inhibition exceeded 90%. (**B and C**) Determination of the minimum fungicidal concentration (MFC) and quantification of colony-forming units (CFU/mL). (**B**) MFC results for brilacidin, with fungicidal activity indicated by the absence of fungal growth (*). (**C**) Represents CFU/mL counts under treatment with increasing compound concentrations. (**D**) Represents the cytotoxicity profile of brilacidin using murine RAW 264.7 macrophages. Untreated cells served as a viability control, and lactate dehydrogenase (LDH)-treated cells as a positive control for cell death. Dashed columns indicate the MIC for each compound, and the dotted line marks the 50% cell viability threshold. Statistical significance was calculated relative to the LDH control, with **** indicating *P* < 0.0001.

For the reference strains, *C. neoformans* H99 and *C. deuterogattii* R265, brilacidin showed minimum inhibitory concentration (MIC) value of 2.5 μM (3.482 µg/mL) (Fig. 1A and Table 1). This MIC confirmed the findings by our group during the screening of Medicines for Malaria Venture (MMV) drug collections (13) and those reported by Dos Reis et al. and Diehl et al. (17, 18). At this concentration, we confirmed that brilacidin completely inhibited fungal growth. To test the consistency of the results, additional strains of *C. neoformans* (*n* = 8) and *C. deuterogattii* (*n* = 7) were investigated and displayed MIC values ranging from 2.5 to 5 μM, as shown in Table 1 (18). The compound was also tested against other pathogenic fungi. No antifungal activity was detected against *Aspergillus fumigatus* isolates, in contrast to the findings reported by Dos Reis et al. (14). However, this discrepancy may be attributed to the higher concentrations used in their study (above 8 μM) compared with those tested here, where the maximum concentration was 10 μM. Additionally, other clinically relevant species, including *Sporothrix schenckii, Sporothrix brasiliensis*, and *Candida albicans*, which had not been tested before, were included in the analysis. Brilacidin did not exhibit antifungal activity against any of these fungi. Collectively, these results suggest that brilacidin displays a more selective antifungal effect toward *Cryptococcus* species and may require higher concentrations to inhibit other fungal pathogens.

**TABLE 1:** MICs of brilacidin against different fungal species and isolates.

Fungal isolates	MIC brilacidin	
	µM	µg/mL
*Cryptococcus* spp.	2.5	3.48
*C. neoformans* H99	2.5	3.48
*C. neoformans* Cn161	2.5	3.48
*C. neoformans* Cn186	2.5	3.48
*C. neoformans* Cn216	2.5	3.48
*C. neoformans* Cn271	2.5	3.48
*C. neoformans* Cn115	5.0	6.96
*C. neoformans* Cg366	5.0	6.96
*C. neoformans* Kn99alpha	5.0	6.96
*C. neoformans* B3501	2.5	3.48
*C. deuterogattii* R265	2.5	3.48
*C. deuterogattii* Cg158	5.0	6.96
*C. deuterogattii* Cg188	2.5	3.48
*C. deuterogattii* Cg221	2.5	3.48
*C. deuterogattii* Cg367	2.5	3.48
*C. deuterogattii* Cg365	2.5	3.48
*C. deuterogattii* Cg460	2.5	3.48
*C. deuterogattii* Cg461	2.5	3.48
*Aspergillus fumigatus*		
*A. fumigatus* Ku80	>20	>27.84
*A. fumigatus* af1163	>20	>27.84
*A. fumigatus* af293	>20	>27.84
*Sporothrix* spp.		
*S. schenckii* 1099-18	20	27.84
*S. brasiliensis* 5110	>20	>27.84
*Candida albicans*		
*C. albicans* OD1	>20	>27.84
*C. albicans* OD10	>20	>27.84
*C. albicans* OD7	>20	>27.84
*C. albicans* OD14	>20	>27.84
*C. albicans* Sc5314	>20	>27.84
*C. albicans* Ca90028	>20	>27.84

Minimum fungicidal concentration (MFC) assays revealed fungicidal activity of brilacidin against *C. neoformans* H99 and *C. deuterogattii* R265, previously characterized by Dos Reis et al. (18), which was confirmed in our study, with MFC values equivalent to the MIC (2.5 μM, 3.482 µg/mL), as confirmed by colony-forming units (CFU) counts (Fig. 1B and 1C). These results demonstrate the reproducibility of the fungicidal activity of brilacidin in independent tests conducted by various laboratories.

Cytotoxicity was assessed in RAW 264.7 macrophages via LDH release. Brilacidin showed an IC_50_ of 40 μM and a selectivity index (SI = IC_50_/MIC) of 16, a value considered favorable for antifungal specificity (19). At the MIC, brilacidin induced only 10.8% cytotoxicity, close to that of untreated controls (6.5%) (Fig. 1D). This low toxicity is consistent with findings by Dos Reis et al., who reported that concentrations of 40 μM and 80 μM did not cause toxic effects in A549 pulmonary cells (18). They also attest to the low toxicity of brilacidin against different cell types.

### Synergistic activity of brilacidin and AmB

**Fig 2:**
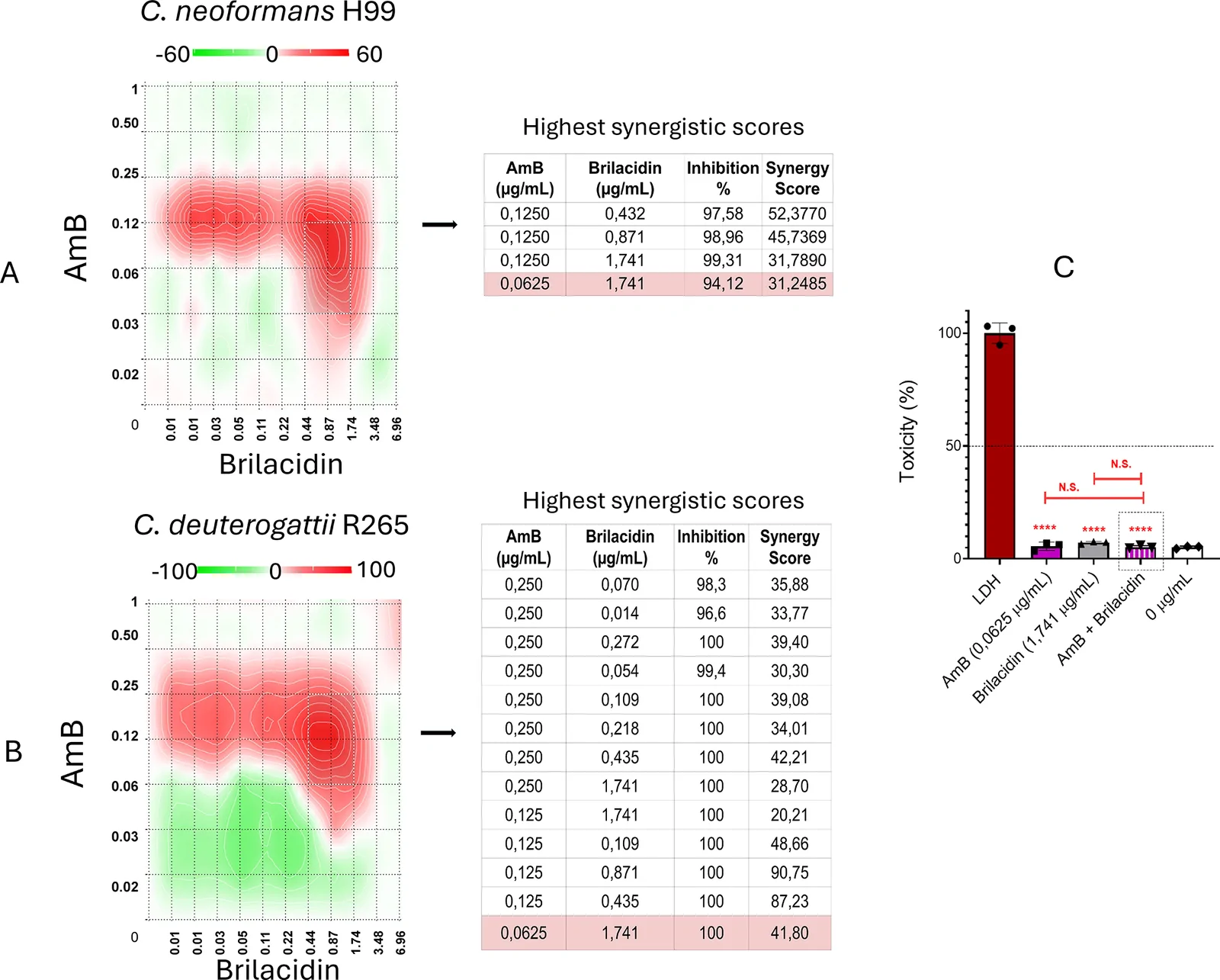
Synergistic activity and toxicity profile of AmB in combination with brilacidin against *C. neoformans* H99 and *C. deuterogattii* R265. Synergy analyses were performed using the SynergyFinder software and the Bliss independence model, based on growth inhibition percentages. Heatmaps represent synergy (red) and antagonism (green). The AmB-brilacidin combination was tested against (**A**) *C. neoformans* H99 and (**B**) *C. deuterogattii* R265. (**C**) Cytotoxicity profile of the AmB-brilacidin synergistic combination (0.0625 µg/mL AmB + 1.741 µg/mL brilacidin) and individual treatments in RAW 264.7 murine macrophages. Cell viability was assessed via LDH release; the dotted line marks 50% viability. Statistical significance was determined in comparison to the positive control (LDH), with **** indicating *P* < 0.0001 and N.S. denoting non-significant differences.

The potential synergistic interaction between AmB and brilacidin was evaluated using SynergyFinder and the Bliss independence model. Brilacidin in combination with AmB showed overall additive effects, with synergy scores of 5.16 for *C. neoformans* H99 and 6.18 for *C. deuterogattii* R265. These results were previously shown using the FIC index method by Diehl et al. against *C. neoformans* (17). However, by using the Bliss independence model, our results showed that specific concentration ranges revealed strong synergistic effects. AmB alone exhibited an MIC of 0.5 µg/mL against both *C. neoformans* H99 and *C. deuterogattii* R265; however, when combined with brilacidin, the effective concentration was reduced. For *C. neoformans* H99, the combination of AmB 0.125 µg/mL and brilacidin 0.3125 µM yielded a synergy score of 57.38. Furthermore, AmB 0.0625 µg/mL combined with brilacidin 1.25 µM achieved a synergy score of 31.25, showing a relevant reduction in the AmB dose. In *C. deuterogattii* R265, the same combination (AmB 0.0625 µg/mL and brilacidin 1.25 µM) produced a synergy score of 41.80 (Fig. 2A and 2B). Our results indicate that this combination may reduce the effective AmB concentration for anti-cryptococcal activity by up to eightfold.

The most promising synergistic condition (AmB 0.0625 µg/mL and brilacidin 1.25 µM) was further evaluated for cytotoxicity in RAW 264.7 macrophages using the LDH release assay. The combination resulted in 5.2% cytotoxicity, comparable to the untreated control (5.1%) and below 10%. No significant differences were observed between the drugs alone and in combination, confirming that the synergistic regimen maintains a favorable safety profile (Fig. 2C).

### Effects of brilacidin on *Cryptococcus* morphology

The morphological effects of brilacidin on *C. neoformans* H99 and *C. deuterogattii* R265 were examined using scanning electron microscopy (SEM), confocal fluorescence microscopy, and transmission electron microscopy (TEM). Morphological analyses were performed to assess structural alterations associated with antifungal activity, with particular emphasis on treatment effects on the cryptococcal capsule, a key virulence factor. Of note, only TEM and optical microscopy have been previously used to evaluate how brilacidin affects *Cryptococcus* (17), and some of these effects were reexamined and/or expanded here.

**Fig 3:**
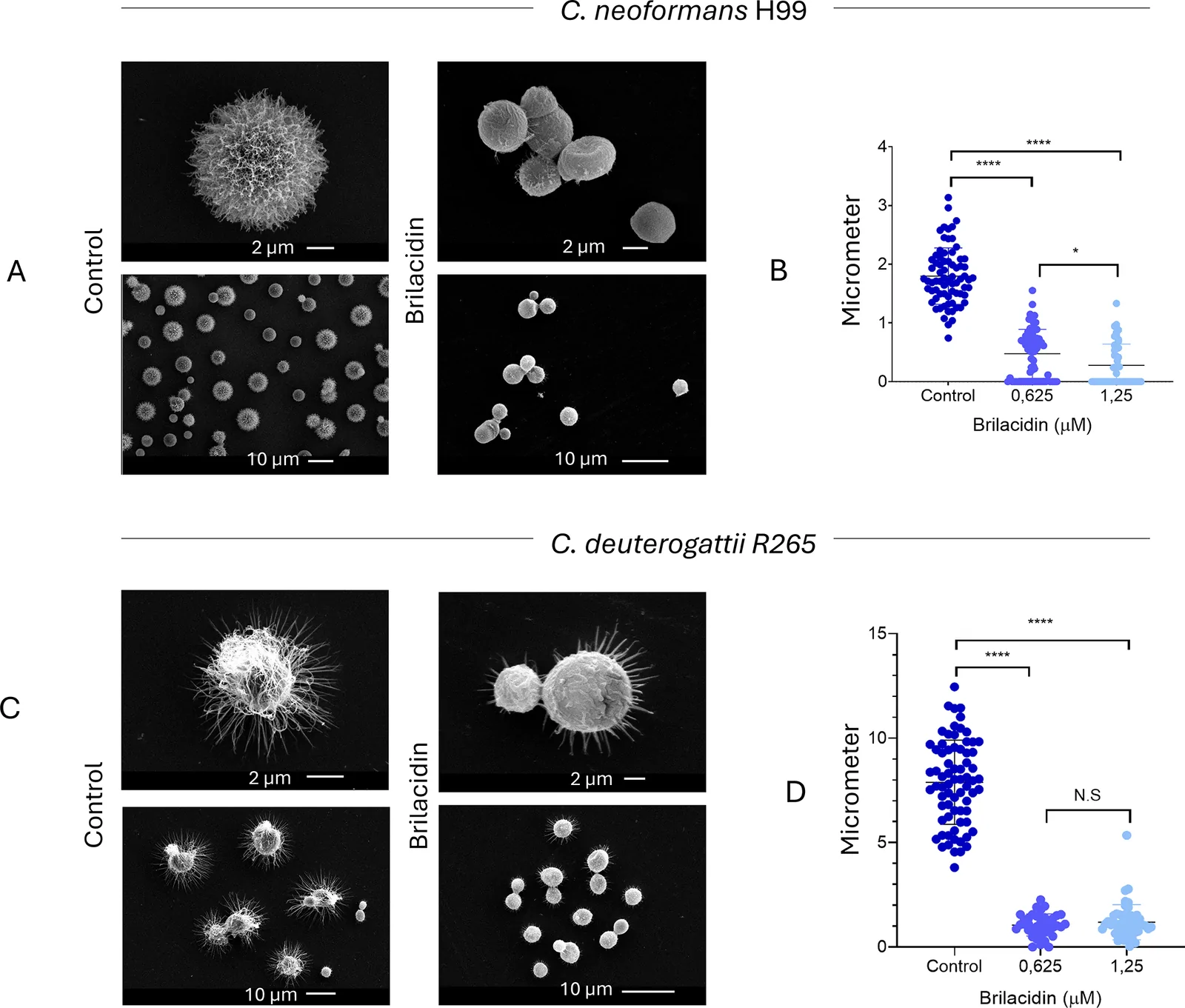
SEM analysis of *C. neoformans* H99 and *C. deuterogattii* R265 cells after treatment with brilacidin. (**A**) Representative SEM images of *C. neoformans* H99 cells untreated (control, 1% DMSO) or treated with brilacidin (0.625 µM). (**B**) SEM images of *C. deuterogattii* R265 cells under the same treatment conditions. (C, D) Quantitative analysis of capsule size (µm) in *C. neoformans* H99 (**C**) and *C. deuterogattii* R265 (**D**) cells. Statistical significance: *P < 0.1, ****P < 0.0001, and N.S., not significant.

SEM analysis at sub-inhibitory concentrations (0.625 µM, 24 h) showed that brilacidin induced pronounced morphological alterations in both species, including loss of capsule integrity, irregular and collapsed cell surfaces, and marked disorganization of capsular fibers (Fig. 3A and C). This observation was corroborated by capsule size measurements, which revealed a significant reduction in capsule thickness in brilacidin-treated cells in both species, in a dose-dependent manner (Fig. 3B and D). Notably, this finding differs from a report by Diehl et al., who found no change in capsule size after brilacidin treatment, even at a higher concentration (1.25 µM) (17).

**Fig 4:**
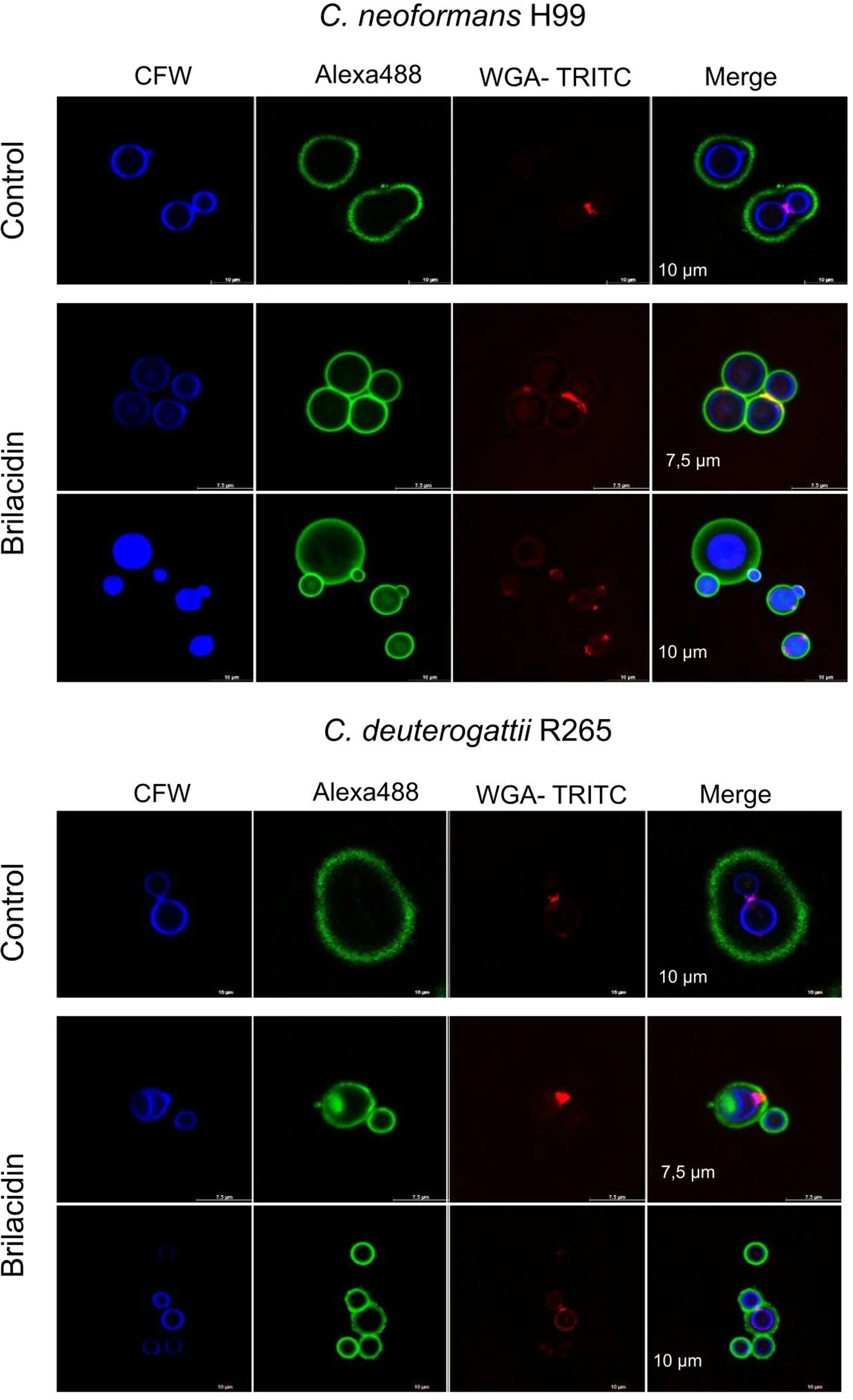
Confocal fluorescence microscopy of *C. neoformans* H99 and *C. deuterogattii* R265, untreated (1% DMSO) or treated with brilacidin. In all panels, chitin in the cell wall was stained with Calcofluor White (CFW; blue), capsule fibers were labeled with Alexa Fluor 488-conjugated monoclonal antibody 18B7 (green), and chitin oligomers were visualized with Wheat Germ Agglutinin conjugated to TRITC (WGA-TRITC; red). The final merged images highlight morphological differences between control and treated cells.

Confocal fluorescence microscopy corroborated the SEM findings. Brilacidin treatment resulted in irregular chitin distribution, capsule reduction, and enhanced staining of chitin oligomers (Fig. 4).

**Fig 5:**
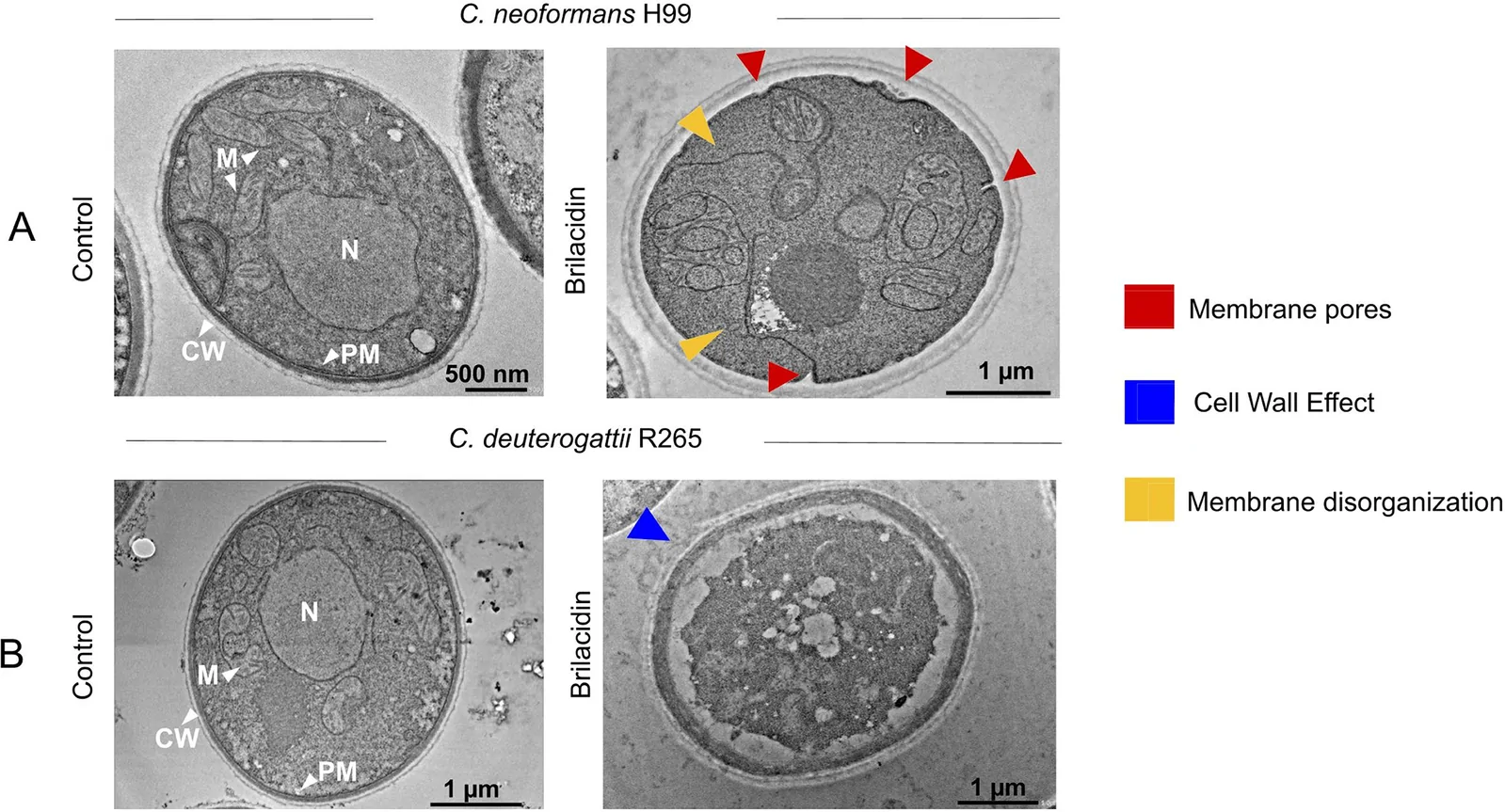
Ultrastructural analysis of *C. neoformans* H99 and *C. deuterogattii* R265 under brilacidin treatment. TEM images of *C. neoformans* H99 (**A**) and *C. deuterogattii* R265 (**B**) under control conditions and after treatment with brilacidin (2.5 µM for 2 h, minimum inhibitory concentration). Key cellular structures are labeled: PM, plasma membrane; M, mitochondria; N, nucleus; CW, cell wall. For both compounds, treated cells exhibit prominent morphological alterations compared to controls. Red arrows indicate membrane pore formation; yellow arrows denote intracellular membrane disorganization; blue arrows highlight the cell wall damage.

TEM analyses at the MICs (2.5 µM, 2 h) revealed that brilacidin disrupted plasma membrane integrity, promoted pore formation, and caused extensive cytoplasmic disorganization and cell damage. These effects were especially pronounced in *C. deuterogattii* (Fig. 5A and B). Our results are consistent with those reported by Diehl et al., who demonstrated that *Cryptococcus* mutants in genes involved in ergosterol and sphingolipid biosynthesis have increased susceptibility to brilacidin. Furthermore, using *Saccharomyces cerevisiae* as a model, the same study showed that brilacidin affected cell membrane organization (17), which may be related to the membrane disruption observed in our study. Moreover, Diehl et al. also reported that treatment of *C. neoformans* with 25 µM brilacidin led to a significant reduction in the inner layer of the fungal cell wall, a phenotype that was not observed in our study.

Altogether, these results demonstrate that brilacidin exerts a strong and broader impact on *Cryptococcus* morphology, affecting capsule structure, cell wall organization, and intracellular integrity.

### Antifungal activity against *C. neoformans* biofilms

**Fig 6:**
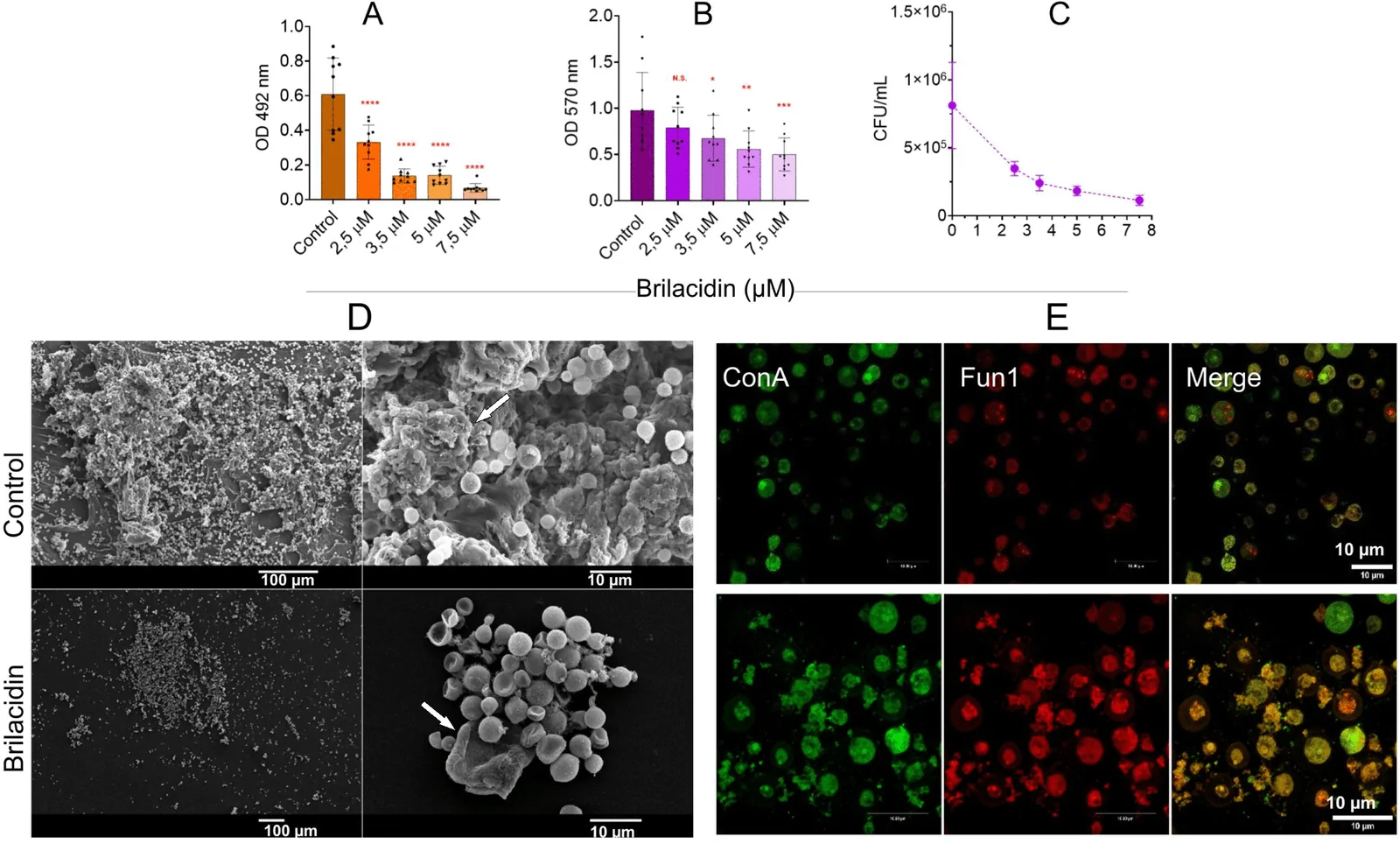
Evaluation of brilacidin’s anti-biofilm activity against *C. neoformans* B3501. (**A**) SEM of biofilms under control and brilacidin treatment (7.5 µM for 24 h). White arrows indicate the presence of extracellular matrix, which is disrupted in treated samples. (**B**) Confocal Fluorescence Microscopy images of biofilms stained with concanavalin A (ConA) to visualize biofilm matrix and FUN-1 to assess cell viability. (**C**) Metabolic activity measured by XTT reduction assay. (**D**) Total biomass quantified by crystal violet staining. (**E**) Quantification of viable cells (CFU/mL) from 72 h mature biofilms treated for 24 h with increasing concentrations of brilacidin (0, 2.5, 3.5, 5, and 7.5 µM). For all assays, treatments were applied for 24 h to pre-formed 72 h biofilms, and untreated controls were exposed to 1% DMSO. Asterisks indicate statistical significance (*P  < 0.1, **P  <  0.01, ***P  <  0.001); N.S., not significant.

The antifungal effects of brilacidin against cryptococcal biofilms were not tested before. The antifungal activity of brilacidin in this model was assessed in biofilms formed by *C. neoformans* B3501, a strain known to generate mature and structured biofilms with a dense extracellular matrix (20). Brilacidin significantly reduced the biofilm’s metabolic activity, as measured by 2,3-bis(2-methoxy-4-nitro-5-sulfophenyl)-2H-tetrazolium-5-carboxanilide (XTT) reduction, with a ~46% decrease at its MIC (2.5 μM, *P* < 0.0001) and up to 90% at 7.5 μM (Fig. 6A). Consistently, total biomass quantification by crystal violet staining revealed that brilacidin reduced biofilm biomass by 20% at 2.5 μM and 49% at 7.5 μM (*P* < 0.001) (Fig. 6B).

To further corroborate these findings, viable cell counts demonstrated that brilacidin decreased biofilm cell density from 8.1 × 10⁵ CFU/mL in untreated control to 1.1 × 10⁵ CFU/mL at 7.5 μM, reinforcing its activity against biofilm-embedded cells (Fig. 6C). The combination of AmB (0.0625 μg/mL) and brilacidin (1.25 μM), although synergistic against planktonic cells, resulted in only partial inhibition of biofilm activity, with a 38% reduction in metabolic activity (*P* < 0.0001) and no significant decrease in total biomass, indicating that anti-biofilm effects are concentration-dependent (data not shown).

Morphological analyses supported these results. SEM revealed that control biofilms displayed intact fungal communities embedded within a dense polysaccharide matrix, whereas brilacidin treatment (7.5 μM, 24 h) led to a marked reduction in cell density, loss of extracellular matrix, and disrupted cellular structures (Fig. 6D). Confocal fluorescence microscopy using concanavalin A (ConA) and 2-chloro-4-(2,3-dihydro-3-methyl-(benzo-1,3-thiazol-2-yl)-methylidene)-1-phenylquinolinium iodide (FUN-1) staining further corroborated these alterations. ConA stains polysaccharides in the cell wall and extracellular matrix (green), whereas FUN-1 labels metabolically active cells by forming red punctate intracytoplasmic structures, and our results showed compromised cell morphology, leakage of intracellular contents, and loss of viability, in sharp contrast to the intact and metabolically active cells in control biofilms. Leakage of intracellular contents was inferred from the increased presence of cellular debris observed by SEM and confocal microscopy, with stained particles in confocal images indicating their cellular origin (Fig. 6E).

### Proteomic analysis of *Cryptococcus* spp. after treatment with brilacidin

The molecular responses of *Cryptococcus* to brilacidin are unknown. Therefore, the proteomic profiles of *C. neoformans* H99 and *C. deuterogattii* R265 were analyzed after 2 h of exposure at the MIC (2.5 µM). Comparative analysis of total proteins revealed treatment-specific differences. The complete list of proteins detected is available in Table S1.

**Fig 7:**
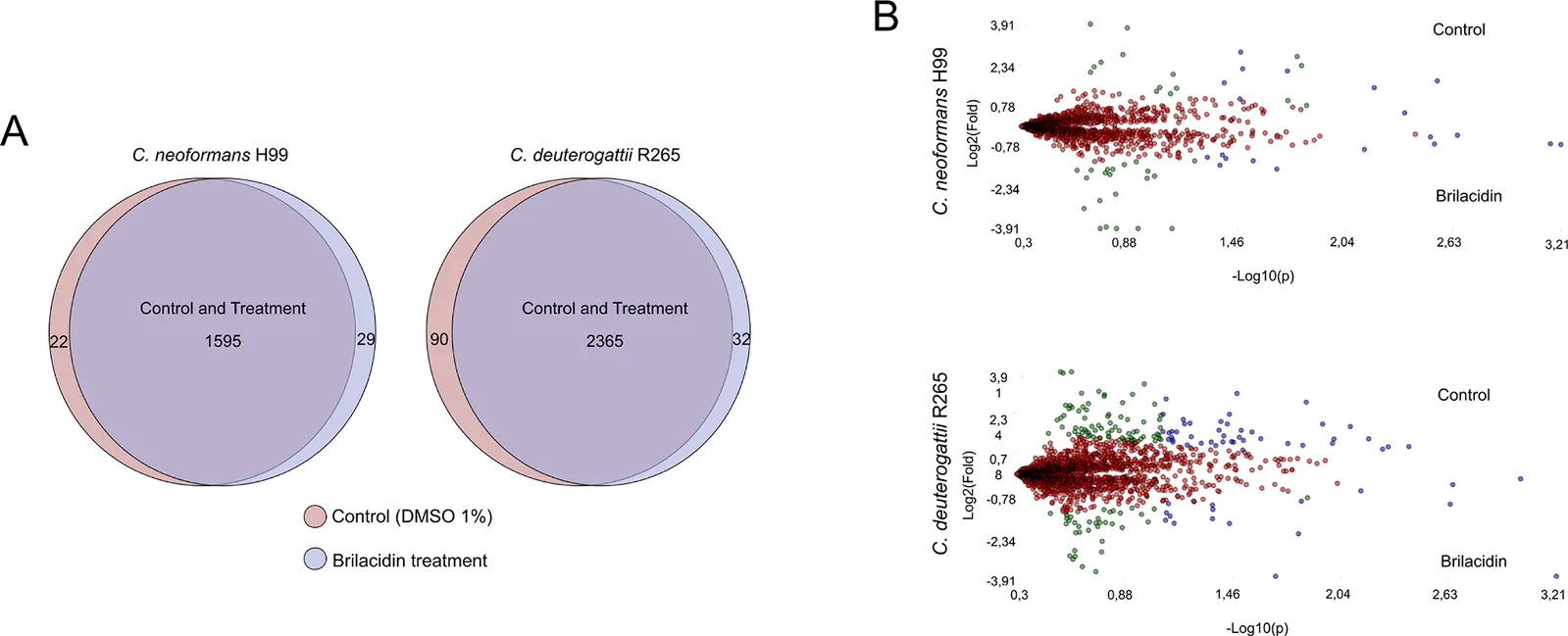
Comparative proteomic analysis of *C. neoformans* and *C. deuterogattii* following brilacidin treatment. (**A**) Venn diagrams showing the overlap of proteins identified in control samples (1% DMSO) and those treated with brilacidin (2.5 µM for 2 h). Only proteins detected in at least two replicates were considered. The diagrams display proteins uniquely identified in the control condition (pink), unique to the brilacidin-treated group (blue), and those shared between both conditions (purple). In *C. neoformans* H99, 22 proteins were uniquely identified in the control group, 29 in the treated group, and 1,595 were common. In *C. deuterogattii* R265, 90 proteins were exclusive to the control, 32 to the treatment, and 2,365 were shared. (**B**) Volcano plots illustrating differentially abundant proteins in response to brilacidin treatment in *C. neoformans* H99 and *C. deuterogattii* R265. The analysis was performed using the T-Fold module in PatternLab for proteomics V (http://www.patternlabforproteomics.org/) with an FDR threshold (Benjamini-Hochberg q-value) of 0.05 and F-stringency of 0.43. Proteins in red did not meet the thresholds for fold change or q-value. Green dots represent proteins with sufficient fold change but non-significant q-values. Blue dots indicate proteins with statistically significant differential abundance (meeting both fold change and q-value criteria).

Brilacidin exposure led to 29 unique proteins in *C. neoformans* (not detected in controls), while 22 were exclusive to untreated control samples. In *C. deuterogattii*, 32 proteins were unique to brilacidin treatment and 90 to controls (Fig. 7A and Table S1). In addition, brilacidin modulated 18 proteins in *C. neoformans* (7 downregulated, 11 upregulated) and 27 in *C. deuterogattii* (20 downregulated, 7 upregulated) (Fig. 7B and Table S1).

**Fig 8:**
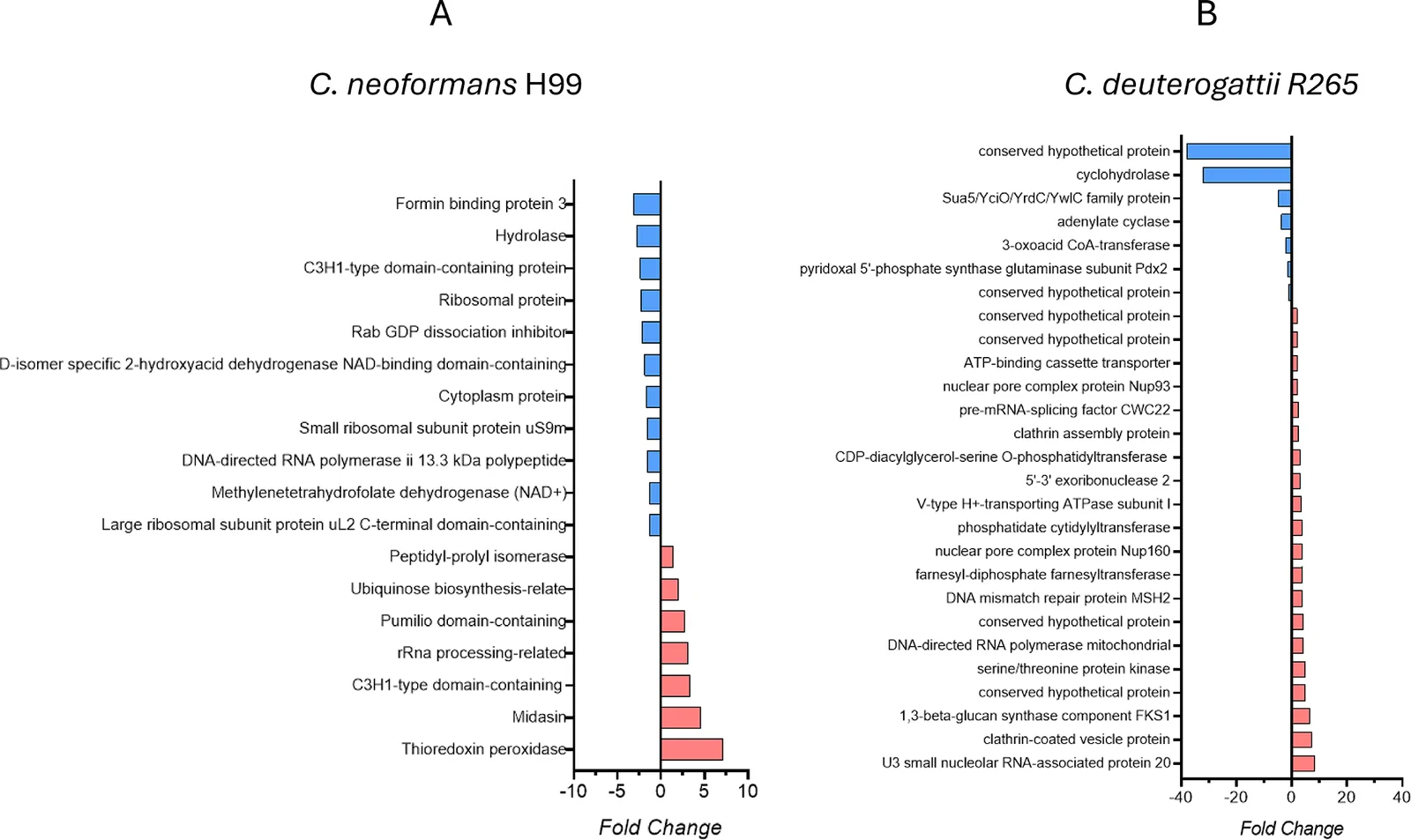
Differentially abundant proteins in *C. neoformans* H99 (**A**) and *C. deuterogattii* R265 (**B**) following treatment with brilacidin. Bar plots show proteins significantly altered in abundance relative to untreated controls; pink bars indicate upregulated proteins and blue bars downregulated proteins.

In *C. neoformans*, brilacidin treatment resulted in 18 differentially abundant proteins, including 11 downregulated and 7 upregulated (Fig. 8A). Upregulated proteins were mainly associated with redox balance and protein folding, such as thioredoxin peroxidase, peptidyl-prolyl isomerase, and midasin, while several ribosomal and metabolic enzymes showed reduced abundance, suggesting a potential suppression of translational and metabolic activity. In *C. deuterogattii*, brilacidin caused broader proteomic alterations, with 27 differentially abundant proteins (7 downregulated, 20 upregulated) (Fig. 8B). The downregulated group included numerous metabolic enzymes (e.g., cyclohydrolase, adenylate cyclase, and 3-oxoadipate CoA-transferase), indicating strong interference in cellular metabolism.

**Fig 9:**
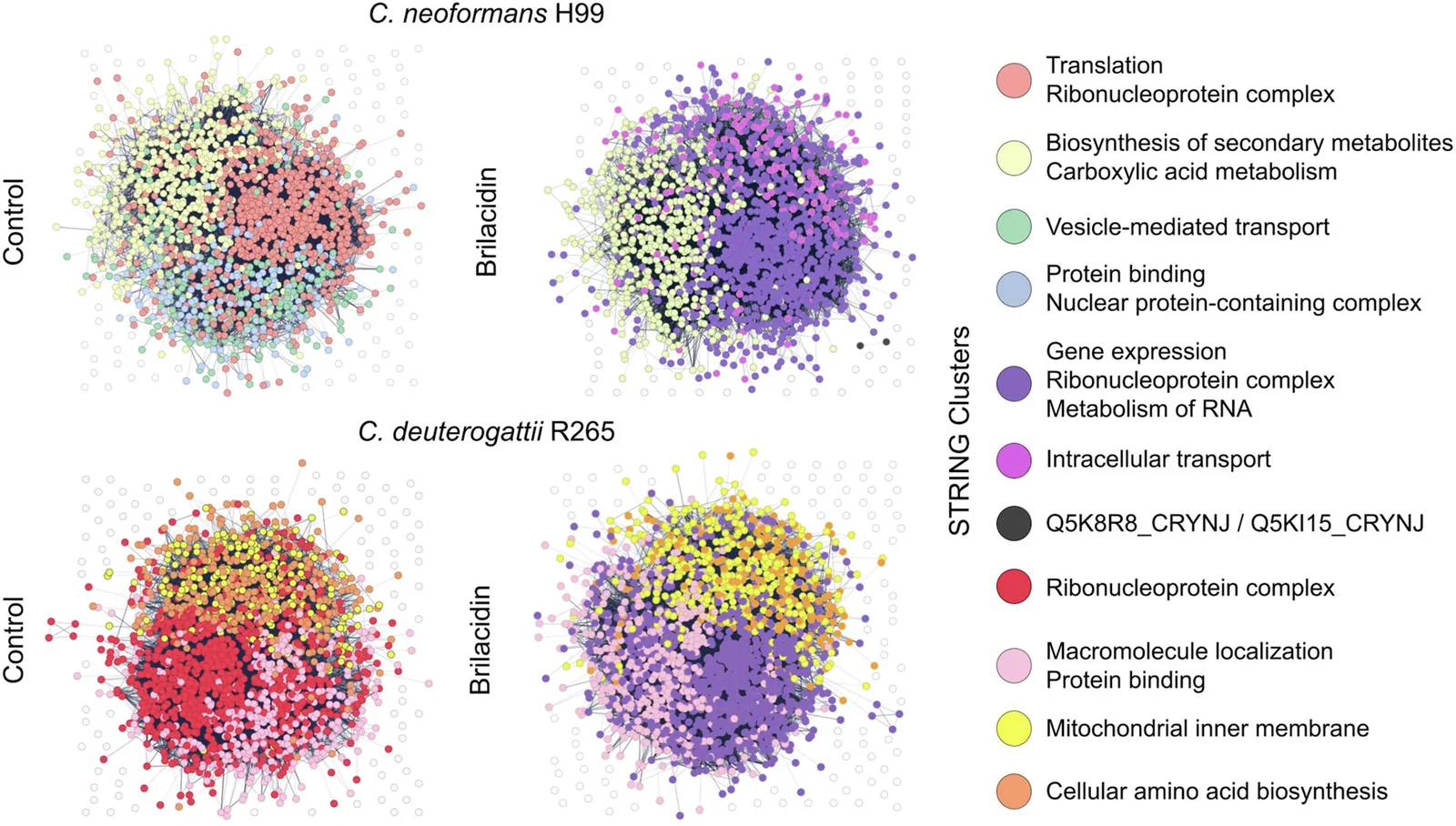
Protein-protein interaction networks in *C. neoformans* H99 and *C. deuterogattii* R265 under brilacidin treatment and control conditions. Protein interaction networks were constructed using all proteins identified in control samples (1% DMSO) and those treated with brilacidin (2.5 µM for 2 h). Each node represents a protein, and edges indicate predicted functional associations. Proteins were grouped into four functional clusters based on k-means clustering analysis using the STRING database. Clusters are color-coded and labeled according to the original functional categories provided by STRING.

Theoretical protein-protein interaction network analysis using STRING demonstrated marked reorganization of functional clusters in both species after drug exposure. In *C. neoformans*, brilacidin treatment abolished a control-associated cluster related to nuclear complexes and protein binding, while promoting new clusters linked to gene expression, RNA metabolism, and ribonucleoprotein complexes. Similar remodeling was observed in *C. deuterogattii* (Fig. 9).

**Fig 10:**
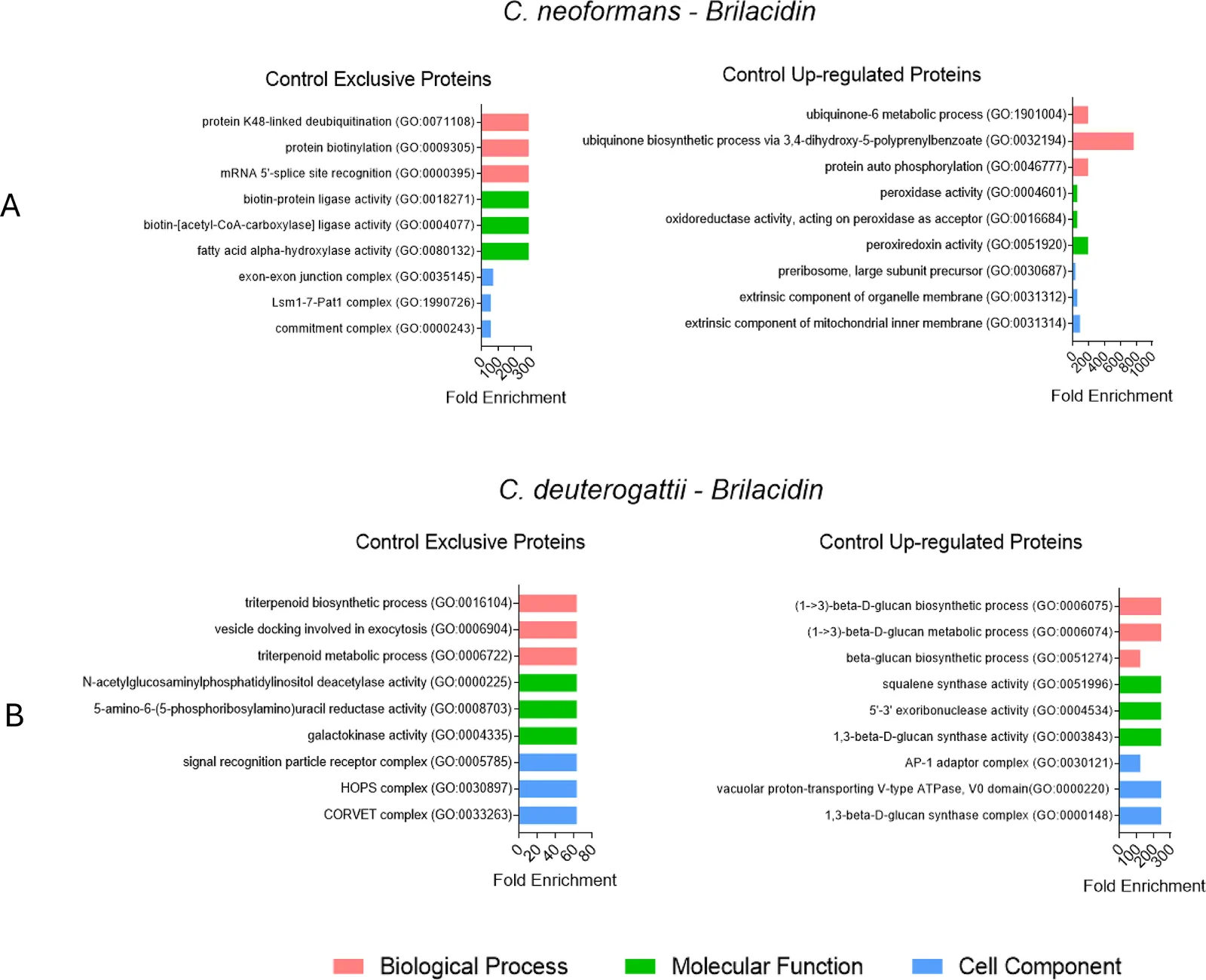
Functional enrichment analysis of proteins affected by brilacidin treatments in *C. neoformans* and *C. deuterogattii*. Gene Ontology (GO) enrichment analysis was used to identify overrepresented biological processes, molecular functions, and cellular components among proteins exclusive to control conditions (left panels) and control-upregulated proteins (right panels) following exposure to brilacidin. The top three enriched GO terms per category are shown based on fold enrichment. (**A**) *C. neoformans* H99 treated with brilacidin: control-exclusive proteins were mainly involved in post-translational modifications (e.g., protein deubiquitination, biotinylation) and fatty acid metabolism, whereas control-upregulated proteins were enriched in oxidative and mitochondrial metabolic processes, including ubiquinone biosynthesis and peroxidase activity. (**B**) *C. deuterogattii* R265 treated with brilacidin: control-exclusive proteins were related to vesicle trafficking (CORVET and HOPS complexes) and triterpenoid and galactose metabolism, while control-upregulated proteins were strongly enriched for β-glucan biosynthesis and vacuolar ATPase and squalene synthase activities, indicating alterations in cell wall and membrane biosynthesis. Bars represent fold enrichment of GO terms classified as Biological Process (pink), Molecular Function (green), or Cellular Component (blue).

Functional enrichment analyses highlighted that brilacidin treatment induced distinct proteomic responses between the two species. In *C. neoformans*, control-exclusive proteins were mainly involved in post-translational modifications such as protein deubiquitination and biotinylation, as well as fatty acid biosynthesis, suggesting these processes were suppressed upon drug exposure (Fig. 10A). In *C. deuterogattii*, brilacidin affected proteins linked to intracellular trafficking and triterpenoid and galactose metabolism, pointing to interference with lipid biosynthesis and membrane homeostasis (Fig. 10B). Among the proteins reduced in abundance after treatment, *C. neoformans* showed enrichment in oxidative and mitochondrial metabolic processes, including ubiquinone biosynthesis and peroxidase activity—consistent with oxidative stress responses—while C. *deuterogattii* displayed strong effects on β-glucan biosynthesis, vacuolar ATPase activity, and squalene synthase, highlighting disruptions in cell wall integrity and sterol metabolism (Fig. 10A and C).The mass spectrometry proteomics data have been deposited in the ProteomeXchange Consortium via the PRIDE (21) partner repository with the dataset identifier PXD070309.

## DISCUSSION

Our work built upon two previous studies. First, brilacidin was characterized in detail by Diehl and colleagues (17) as an anti-*Cryptococcus* compound with significant therapeutic potential *in vitro* and *in vivo*. In addition, brilacidin was identified by our group, within a compound library, as a potent inhibitor of *C. neoformans* growth (13). In the present study, we addressed questions that had remained insufficiently explored in these earlier investigations, with the aim of advancing the validation of brilacidin as an anti-cryptococcal agent. In this sense, we combined biofilm inhibition, phenotypic, microscopic, and proteomic approaches to better understand how brilacidin affects *Cryptococcus*.

In this investigation and in a previous study, brilacidin demonstrated consistent and potent antifungal activity against all tested isolates of *C. neoformans* and *C. deuterogattii*, with MICs between 2.5 and 5 μM. Its spectrum appeared narrower, since higher MICs were observed for *Aspergillus*, *Candida*, and *Sporothrix* species, in line with previous studies indicating preferential activity against *Cryptococcus (*17, 22, 23). Brilacidin is a host-defense peptide mimetic with documented antibacterial, antiviral, and antifungal activity, currently in Phase II clinical trials for bacterial infections, oral mucositis, and COVID-19 (17, 18, 24–27). Its low cytotoxicity, with a selectivity index above 10, further reinforces its safety profile and specificity for fungal targets.

Importantly, brilacidin enhanced the activity of AmB, reducing its MIC by up to eightfold in both *Cryptococcus* species. The ability of brilacidin to potentiate AmB was in accordance with the study by Diehl et al. (17). The observed synergism was confirmed across different concentration combinations and achieved high inhibition levels with minimal cytotoxicity. Such an approach could allow dose reduction of amphotericin B, mitigating its toxicity while maintaining efficacy, which is particularly relevant in the context of cryptococcosis treatment. This is consistent with previous reports of synergism not only with AmB but also with other antifungals, supporting its potential use in combination therapy (17, 18, 23). For example, its synergistic activity with caspofungin, which targets complementary cellular processes, was observed by Reis et al. and Diehl et al. against *A. fumigatus*, *C. albicans*, *C. auris*, and *C. neoformans* (17, 18) and represents a promising antifungal strategy. A more recent study shows that brilacidin also acts in synergism with ibrexafungerp, inhibiting the growth of *A. fumigatus* voriconazole- and caspofungin-resistant clinical isolates (23).

Our analyses of cellular responses confirmed that brilacidin primarily targets the fungal membrane, promoting pore formation, structural disorganization, and altered sterol and lipid profiles (17). Our proteomic analyses corroborated these findings, showing decreased abundance of proteins involved in sterol and fatty acid biosynthesis. Ergosterol, the main fungal sterol, is essential for membrane integrity, virulence, and cell regulation; disruption of its biosynthesis represents the classical mechanism of action of polyenes and azoles (28–30). Thus, brilacidin appears to interfere with this pathway, contributing to its fungicidal activity.

These membrane-disrupting effects are consistent with other studies. In gram-positive bacteria, such as *Staphylococcus aureus*, it has been shown that brilacidin targets the cell membrane, causing depolarization and inducing cell wall stress (31). Similarly, Diehl et al. (17) suggested that in *C. neoformans*, brilacidin’s activity is closely linked to ergosterol distribution and organization within the fungal membrane, as previously mentioned in this manuscript. Their results indicated that membrane disruption compromises organization and permeability, ultimately leading to cell death. Furthermore, experiments with *Cryptococcus* mutants revealed that defects in genes involved in ergosterol and sphingolipid biosynthesis increased susceptibility to brilacidin, with MIC values as low as 0.62 μM (17). An effect on membrane potential was also reported for *A. fumigatus* (18).

Beyond membrane disruption, brilacidin also affected the cell wall, as previously observed by Diehl et al. (17). Microscopy revealed cell wall damage in both *C. neoformans* and *C. deuterogattii*, while proteomics indicated reduced abundance of proteins associated with β-1,3-glucan synthesis. Since echinocandins, which target this pathway, are ineffective against *Cryptococcus* (32), brilacidin’s ability to compromise cell wall integrity represents a unique and promising feature.

In our study, brilacidin affected a major cryptococcal virulence factor. Scanning and confocal fluorescence microscopy demonstrated alterations in the polysaccharide capsule, a major determinant of virulence (33, 34), suggesting that brilacidin interferes with capsule formation and architecture. This result contrasts with a previous report that found capsule alterations only when brilacidin was combined with caspofungin (17), suggesting that its impact may depend on strain background or experimental conditions. We attribute these differences to variations in experimental conditions, since in our study we used a capsule-induction medium (10% diluted Sabouraud in 50 mM MOPS [morpholinepropanesulfonic acid], pH 7.4 [35]), in contrast to the use of YPD. Moreover, brilacidin strongly inhibited *Cryptococcus* biofilms, reducing metabolic activity, extracellular matrix production, and viable cell counts in a concentration-dependent manner. Morphological analyses further confirmed biofilm disruption, highlighting brilacidin’s ability to overcome one of the main barriers to antifungal therapy. Similar anti-biofilm effects were also described against *Aspergillus* species, suggesting broader relevance for this activity (18).

Overall, our findings demonstrated the potent antifungal activity of brilacidin, showing fungicidal and selective activity against *Cryptococcus*, with additional effects on membrane and cell wall integrity, capsule formation, biofilm development, as well as synergism with AmB. These multifaceted activities reinforce brilacidin as a promising candidate for the development of new antifungal therapies.

## MATERIALS AND METHODS

### Antifungals

Brilacidin (MMV1634402) was obtained from MMV. The molecule was maintained as stock solutions at 20 mM in DMSO and stored at −20°C.

### Strains and growth conditions

The primary fungal strains used in this study were *Cryptococcus neoformans* H99 and *C. deuterogattii* R265. For the determination of MIC, additional isolates were included: *C. neoformans* Cn161, Cn186, Cn216, Cn271, Cn115, Cg366, B3501, and Kn99alpha; *C. deuterogattii* Cg158, Cg188, Cg221, Cg367, Cg365, Cg460, and Cg46; *Aspergillus fumigatus* Ku80, Af1163, and Af293; *Sporothrix schenckii* 1099-18 and *S. brasiliensis* 5110; and *Candida albicans* OD1, OD10, OD7, and OD14 (clinical isolates from the Geriatric Dentistry Department at Univale, Minas Gerais, Brazil), SC5314 (ATCC MYA-2876), and ATCC 90028. For biofilm experiments, the *C. neoformans* B3501 strain was used. All isolates were obtained from the collection of the Laboratory of Pathogenic Fungi, Instituto Carlos Chagas, Fiocruz-PR. *Cryptococcus* spp. isolates were cultured on Sabouraud dextrose agar plates (2% peptone, 1% yeast extract, 4% dextrose, and 1.5% agar) at 30°C for 48 h. *Candida* spp. isolates were incubated at 30°C for 24 h, and *Aspergillus* spp. at 35°C for 72 h under the same medium. *Sporothrix* spp. isolates were cultured in brain heart infusion (Difco) broth supplemented with 2% glucose for 7 days at 37°C under constant agitation (180 rpm).

### Antifungal susceptibility testing

In all assays, DMSO concentration was 1%, including the controls without any compound treatment. The MIC was determined following the standardized protocol from The European Committee on Antimicrobial Susceptibility Testing (EUCAST), EDef 7.3.1 reference method for yeasts and EDef 9.4 reference method for conidia-forming molds (36, 37). Compounds were tested over a concentration range of 0.019 to 10 μM in Roswell Park Memorial Institute (RPMI) medium (Sigma-Aldrich), supplemented with 2% glucose and buffered to pH 7.0 with 165 mM MOPS.

For inoculum preparation, isolates of *Cryptococcus* spp. were subcultured on Sabouraud dextrose agar at 30°C for 48 h, *Aspergillus fumigatus* at 35°C for 72 h, and *Candida albicans* at 30°C for 24 h. Following incubation, *A. fumigatus* conidia were harvested in sterile water containing 0.1% Tween 20, and suspensions with less than 5% hyphal fragments were standardized based on conidial counts. Inocula of *Sporothrix* spp. were prepared from cultures between the third and fifth passages, selecting only those with hyphal content below 5%. Finally, all fungal suspensions were adjusted to a final concentration of 2.5 × 10⁵ cells/mL in sterile water. The plates were incubated at 35°C for 48 h, and fungal growth was evaluated by measuring optical density (OD) at 530 nm using a Synergy H1M2F microplate reader (BioTek). The MIC was defined as the lowest compound concentration that inhibited ≥90% of fungal growth.

For MFC determination, 5 μL from the previously prepared MIC plate well were plated onto Sabouraud dextrose agar and incubated at 30°C for 48 h. The MFC was defined as the lowest concentration at which no visible fungal growth was observed. Additionally, 10 μL aliquots from the same wells were plated in parallel for CFU quantification.

### Checkerboard assay with AmB

The synergistic activity of the compounds with AmB was determined using the checkerboard assay (38). The initial concentrations were defined based on previously determined MIC values, resulting in a concentration range of 0.009 μM–5 μM for brilacidin and 0.016 μg/mL–1 μg/mL for AmB (Sigma). The assay was conducted in 96-well microplates, where each well received 50 µL of an AmB dilution, 50 µL of a brilacidin dilution, and 100 µL of the fungal inoculum. Fungal suspensions and fungal growth measurements followed the EUCAST protocol previously described. Considering the differences in chemical nature and cellular targets of AmB and the test compounds, the Bliss independence model (39) was used to calculate synergy scores via the SynergyFinder tool (40).

### Cytotoxicity assay

The cytotoxicity assay was performed using the CytoTox 96 Non-Radioactive Cytotoxicity Assay Kit (Promega), employing murine RAW 264.7 macrophages maintained in Dulbecco’s Modified Eagle Medium supplemented with 10% fetal bovine serum. Macrophages were seeded at a density of 10⁵ cells per well and incubated overnight at 37°C in a 5% CO₂ atmosphere. The following day, cells were treated with brilacidin alone or in combination with AmB at synergistic combinations for 24 h. Control wells included cells treated with 1% DMSO (viability control) and cells treated with the lysis solution provided by the manufacturer (cytotoxicity control). The assay was carried out following the manufacturer’s instructions, and cytotoxicity was assessed based on the release of LDH from damaged cells. The selectivity index (SI) was calculated as the ratio of IC₅₀ to MIC, with IC₅₀ defined as the concentration that induced 50% cytotoxicity. A selectivity index greater than 10 was considered indicative of high selectivity for fungal cells.

### Growth curves

Fungal growth analysis was performed using brilacidin concentrations ranging from 5 to 0.625 μM, along with control conditions without treatment. *C. neoformans* H99 and *C. deuterogattii* R265 were previously incubated overnight in liquid YPD medium and then adjusted to a concentration of 5 × 10⁵ cells/mL in capsule-inducing medium (10% diluted Sabouraud in 50 mM MOPS, pH 7.4) (35). The assay was conducted in 96-well microplates and incubated in a Synergy H1M2F plate reader (BioTek). During the kinetic assay, the temperature was maintained at 37°C, and OD at 530 nm was recorded hourly for 49 h.

### SEM

*C. neoformans* H99 and *C. deuterogattii* R265 strains were cultured overnight in liquid YPD medium at 30°C with agitation (200 rpm), washed three times with sterile phosphate-buffered saline (PBS), and the cell suspensions were adjusted to 5 × 10⁴ cells/mL in capsule-inducing medium (35). Control and treated (0.625 μM) cultures were incubated at 37°C for 24 h. Cells were washed three times with sterile PBS and fixed with 2.5% glutaraldehyde in 0.1 M sodium cacodylate buffer (pH 7.4) for 1 h at room temperature. After fixation, the cells were washed three times with post-fixative solution (0.1 M sodium cacodylate buffer, pH 7.2, containing 2 mM magnesium chloride and 0.2 M sucrose in distilled water). The samples were placed onto 0.01% poly-L-lysine pre-treated coverslips to adhere for 1 h at room temperature, followed by dehydration in a graded ethanol series (30%, 50%, and 70% ethanol for 5 min each, 90% ethanol for 10 min, and two final washes in 100% ethanol for 10 min each). The cells were immediately subjected to critical point drying using a Leica EM CPD300, sputter-coated with a thin layer of gold, and analyzed using a JEOL JSM-6010 Plus/LA scanning electron microscope (JEOL) operating at 10 keV.

### TEM

*C. neoformans* H99 and *C. deuterogattii* R265 strains were subcultured in liquid YPD medium and incubated overnight at 30°C under agitation. The fungal suspension was then prepared in liquid YPD at a concentration of 6.5 × 10⁶ cells/mL and incubated at 30°C, 200 rpm for 4 h. Subsequently, control and treated (2.5 μM) cultures were incubated under the same conditions for an additional 2 h.

The cells were washed three times with sterile PBS and fixed for 2 h in a solution containing 2% glutaraldehyde and 4% paraformaldehyde in 0.1 M sodium phosphate buffer (pH 7.4). All subsequent steps were carried out at 4°C. The samples were washed three times with 0.1 M sodium phosphate buffer, and the post-fixation was performed using 1% potassium permanganate in double-distilled water for 1 h, followed by three 5 min washes with double-distilled water. In the next step, 0.15% tannic acid in 0.1 M sodium phosphate buffer (pH 7.4) was applied for 1 min, followed by three washes with double-distilled water.

The samples were then incubated with 2% uranyl acetate for 1 h and again washed three times with double-distilled water, followed by dehydration using a graded ethanol series (50%, 70%, 90%, 95%, and three times of 100%, 10 min each). Following dehydration, samples were infiltrated with Spurr resin in increasing resin-to-ethanol ratios of 1:2, 1:1, and 2:1, for 1 h each, and incubated overnight in 100% Spurr resin. On the following day, infiltration was continued with 100% Spurr resin containing the polymerization catalyst for an additional 4 h. Complete polymerization was achieved by incubating the samples at 60°C for 48 h. The embedded samples were then sectioned and stained with 4% uranyl acetate for 20 min, followed by lead citrate staining.

Finally, the samples were examined using a JEOL 1400 Plus transmission electron microscope (JEOL) operating at 100 kV.

### Confocal fluorescence microscopy

*Cryptococcus neoformans* H99 and *C. deuterogattii* R265 strains were cultured overnight in liquid YPD medium at 30°C with agitation (200 rpm), washed three times with sterile PBS, and adjusted to 5 × 10⁴ cells/mL in capsule-inducing medium (35). Control and treated (0.625 μM) cultures were incubated at 37°C for 24 h.

Following incubation, cells were fixed with 4% paraformaldehyde for 30 min, washed three times with PBS, and blocked with 1% bovine serum albumin (BSA) in PBS for 1 h at 37°C. Cells were then stained for chitin using 12.5 μM CFW (Sigma) in PBS for 30 min at 37°C. Subsequently, fungal cells were incubated with 10 μg/mL of the monoclonal antibody 18B7—which binds to capsular polysaccharide fibers (kindly provided by Dr. Arturo Casadevall)—followed by incubation with a secondary anti-IgG antibody conjugated to Alexa Fluor 488 (Invitrogen) at a 1:1,000 dilution in 1% BSA (200 μL) for 1 h at 37°C. After incubation, the cells were washed three times with PBS, collected, and stained with WGA-TRITC at 5 μg/mL—which binds to chitin oligomers—for 30 min at 37°C.

Finally, the suspensions were washed again and stored at 4°C until analysis using a Leica Stellaris 8 confocal microscope (Leica).

### Effect of brilacidin on *C. neoformans* biofilm

The *C. neoformans* B3501 strain was cultured overnight in liquid YPD at 30°C and 200 rpm. The next day, the suspension was adjusted to 1 × 10⁷ cells/mL in RPMI medium (Sigma-Aldrich) supplemented with 2% glucose and buffered to pH 7.0 with 165 mM MOPS. For biofilm formation, cells were seeded in 96-well plates and incubated at 37°C with 5% CO₂ for 72 h, with fresh RPMI added after 48 h. Treatments were applied at 2.5, 3.5, 5, and 7.5 µM, while controls received 1% DMSO. Plates were incubated for an additional 24 h under the same conditions.

Biofilm metabolic activity was determined by the XTT reduction assay, using 1.72 mg/mL XTT and 1 µL of 1 mM menadione in PBS, followed by 3 h incubation at 37°C and absorbance reading at 492 nm. Biomass quantification was performed by crystal violet staining: after fixation with methanol, biofilms were stained with 1% crystal violet, washed, and destained with 33% acetic acid; absorbance was read at 570 nm.

To assess cell viability, brilacidin-treated biofilms were disrupted in 0.05% Tween-PBS, scraped, and homogenized. Serial dilutions were plated on Sabouraud agar and incubated at 30°C for 48 h to determine CFU/mL. For morphological analysis, biofilms formed on plastic coupons were treated with 7.5 µM brilacidin for 24 h at 30°C and processed for SEM. Confocal fluorescence microscopy was performed on biofilms grown on glass coverslips, stained with concanavalin A (Invitrogen) (15 µM) and FUN-1 (Invitrogen) (10 µM), and visualized using a Leica Stellaris 8 confocal microscope.

### Preparation of fungal samples for protein identification and quantification

*C. neoformans* H99 and *C. deuterogattii* R265 were subcultured in liquid YPD medium and incubated overnight at 30°C with agitation at 200 rpm. Suspensions were adjusted to a concentration of 6.5 × 10⁶ cells/mL in YPD, initially incubated at 30°C under agitation for 4 h and then treated with brilacidin at the MIC for 2 h. A control condition containing only 1% DMSO vehicle was included. Cells were centrifuged and washed three times with sterile PBS. The resulting cell pellets were frozen and stored at −80°C until further processing. This protocol was repeated three times to obtain biological triplicates.

Next, cultures were lyophilized for 18 h at 0.04 mBar and −50°C. Fungal cells were lysed by adding 0.1 mm zirconium beads (1:1 ratio) followed by 10 cycles of vortex agitation for 1 min each, interspersed with 1 min incubations on ice (41). Subsequently, 8 M urea was added to the pellet, and the lysis process was repeated for five cycles under the same conditions. After lysis, samples were centrifuged at 18,000 rpm and 4°C for 15 min. Supernatants were collected, and protein concentrations were determined by fluorometric quantification using a Qubit fluorometer (Thermo Fisher Scientific Inc.).

A total of 100 µg of protein extract was reduced with dithiothreitol (DTT) to a final concentration of 10 mM for 30 min at 56°C. Samples were cooled to room temperature and alkylated with 15 mM iodoacetamide for 25 min in the dark. The solution was then incubated again with 10 mM DTT for 15 min at room temperature in the dark. Protein extracts were diluted with 50 mM ammonium bicarbonate to reduce the urea concentration to 2 M. The pH was verified to be between 7 and 8. Samples were then digested enzymatically with trypsin Gold (Promega) (0.5 µg/µL, 1:50 [wt/wt]) and incubated overnight at 37°C. Following digestion, trifluoroacetic acid was added to a final concentration of 0.5% (pH < 2), and the digested proteins were quantified using fluorometric quantification with a Qubit system (Thermo Fisher Scientific Inc.).

Final sample preparation involved desalting and concentration using Stage-Tip systems. Initially, C18 Empore disks (3M) were carefully inserted into 200 µL pipette tips. The columns were conditioned by adding 100 µL of 100% methanol, followed by centrifugation for 2 min at 1,000 × *g*. Subsequently, 100 µL of 0.1% formic acid was added under the same centrifugation conditions. Then, 200 µL of the prepared samples were loaded onto the column and centrifuged until complete passage. Finally, the columns were washed with 200 µL of 0.1% formic acid in five consecutive cycles.

### Mass spectrometry

The analysis was performed using an Ultimate 3000 nano-liquid chromatography system (Thermo Fisher Scientific Inc.). The previously desalted peptides, analyzed in technical duplicates, were loaded onto reversed-phase columns (30 cm in length and 75 μm inner diameter) packed with ReprosilPur C18 Aqua stationary phase (Dr. Maisch), consisting of 3 μm particles with a pore size of 120 Å. Peptide elution was achieved using a 120 min chromatographic gradient, ranging from 5% to 40% mobile phase B (95% acetonitrile in 0.1% formic acid). The eluate was directed into an Orbitrap Fusion Lumos mass spectrometer (Thermo Fisher Scientific Inc.).

Mass spectra acquisition on the Orbitrap Fusion Lumos was performed in data-dependent acquisition mode, alternating automatically between full MS scans and MS/MS scans, with a dynamic exclusion window of 45 s. Full MS scans were acquired at a resolution of 60,000 at m/z 200, with each cycle lasting 2 s. The most intense ions with charge states of 2+ and 3+ were isolated and fragmented by higher-energy collisional dissociation using a normalized collision energy of 30. The chromatographic gradient and instrument functions were controlled using Xcalibur 4 software (Thermo Fisher Scientific Inc.). Operational parameters of the mass spectrometer included a spray voltage of 2.6 kV, capillary temperature maintained at 250°C, absence of auxiliary or sheath gas flow, and automatic gain control enabled. The S-lens radio frequency level was set to 70%.

### Peptide spectral matching (PSM) and PSM validation

Data analysis was performed using the PatternLab for Proteomics V software (available at: http://www.patternlabforproteomics.org/) (42). The initial step involved obtaining PSMs through the Y.A.D.A. 3.0 algorithm (43), which identifies multiplexed MS2 spectra (44). At this stage, organism-specific protein databases were uploaded into the software. For *C. neoformans* H99, the database was retrieved from the UniProt (available at http://www.uniprot.org/) on 27 August 2024, corresponding to *C. neoformans* var. *neoformans* serotype D (strain JEC21/ATCC MYA-565; organism ID: 214,684). For *C. deuterogattii* R265, the protein data set was obtained from the FungiDB (available at https://fungidb.org/fungidb/app) on the same date, based on *C. gattii* VGII R265. Validation was performed using the Search Engine Processor (45), and protein quantification was carried out based on extracted ion chromatograms. Only proteins identified with at least two unique peptides were considered for analysis; contaminants and decoys were excluded.

### Relative protein quantification, protein-protein interactions, and functional characterization

The final data were analyzed using two modules provided by PatternLab for Proteomics V: the Approximately Area-Proportional Venn Diagram and T-Fold (46). Both analyses were filtered to include results from at least two biological replicates. Additional protein-protein interaction analyses were performed using STRING software (available at https://string-db.org/) (47) on 1 September 2024. Functional enrichment analyses based on Gene Ontology (*P* ≤ 0.05) were performed using the FungiDB platform (available at https://fungidb.org/fungidb/app) (48) on 20 February 2025.

### Statistical analysis

Data were analyzed with GraphPad Prism software (GraphPad Software, San Diego, CA, USA). Statistical significance was defined as *P* < 0.05. Capsule size measurements were evaluated using a one-way ANOVA followed by Tukey’s *post hoc* test. In the figures, ∗, ∗∗, ∗∗∗, and ∗∗∗∗ denote *P*-values <0.1, <0.01, <0.001, and < 0.0001, respectively.

## Data Availability

The mass spectrometry proteomics data have been deposited in the ProteomeXchange Consortium via the PRIDE partner repository with the dataset identifier PXD070309.

## SUPPLEMENTAL MATERIAL

Table S1 spectrum.00138-26-s0001.xlsx List of proteins identified after treatment with brilacidin.

ASM does not own the copyrights to Supplemental Material that may be linked to, or accessed through, an article. The authors have granted ASM a non-exclusive, world-wide license to publish the Supplemental Material files. Please contact the corresponding author directly for reuse.
